# Assessing the Therapeutic Impacts of HAMLET and FOLFOX on BRAF-Mutated Colorectal Cancer: A Study of Cancer Cell Survival and Mitochondrial Dynamics In Vitro and Ex Vivo

**DOI:** 10.3390/medicina60010142

**Published:** 2024-01-12

**Authors:** Justas Žilinskas, Darius Stukas, Aldona Jasukaitienė, Inga Žievytė, Zbigniev Balion, Jurgita Šapauskienė, Rasa Banienė, Henrikas Paužas, Paulius Lizdenis, Vaidotas Čėsna, Žilvinas Dambrauskas, Antanas Gulbinas, Algimantas Tamelis

**Affiliations:** 1 Department of Surgery, Medical Academy, Faculty of Medicine, Lithuanian University of Health Sciences, LT-50161 Kaunas, Lithuania; henrikas.pauzas@lsmu.lt (H.P.); paulius.lizdenis@lsmu.lt (P.L.); vaidotas.cesna@lsmu.lt (V.Č.); zilvinas.dambrauskas@lsmu.lt (Ž.D.); antanas.gulbinas@lsmu.lt (A.G.); algimantas.tamelis@lsmu.lt (A.T.); 2 Institute of Digestive Research, Medical Academy, Faculty of Medicine, Lithuanian University of Health Sciences, LT-50161 Kaunas, Lithuania; darius.stukas@lsmu.lt (D.S.); aldona.jasukaitiene@lsmu.lt (A.J.); inga.zievyte@lsmu.lt (I.Ž.); 3 Preclinical Research Laboratory for Medicinal Products, Institute of Cardiology, Faculty of Pharmacy, Medical Academy, Lithuanian University of Health Sciences, LT-50162 Kaunas, Lithuania; zbigniev.balion@lsmu.lt; 4 Department of Biochemistry, Lithuanian University of Health Sciences, LT-50161 Kaunas, Lithuania; jurgita.sapauskiene@lsmu.lt (J.Š.); rasa.baniene@lsmu.lt (R.B.)

**Keywords:** colorectal cancer, BRAF mutation, HAMLET, bioactive milk compound, mitochondrial function, ex vivo treatment, precision medicine

## Abstract

*Background and Objectives*: Colorectal cancer (CRC) is a major global health challenge. The BRAF V600E mutation, found in 8–12% of CRC patients, exacerbates this by conferring poor prognosis and resistance to therapy. Our study focuses on the efficacy of the HAMLET complex, a molecular substance derived from human breast milk, on CRC cell lines and ex vivo biopsies harboring this mutation, given its previously observed selective toxicity to cancer cells. *Materials and Methods:* we explored the effects of combining HAMLET with the FOLFOX chemotherapy regimen on CRC cell lines and ex vivo models. Key assessments included cell viability, apoptosis/necrosis induction, and mitochondrial function, aiming to understand the mutation-specific resistance or other cellular response mechanisms. *Results:* HAMLET and FOLFOX alone decreased viability in CRC explants, irrespective of the BRAF mutation status. Notably, their combination yielded a marked decrease in viability, particularly in the BRAF wild-type samples, suggesting a synergistic effect. While HAMLET showed a modest inhibitory effect on mitochondrial respiration across both mutant and wild-type samples, the response varied depending on the mutation status. Significant differences emerged in the responses of the HT-29 and WiDr cell lines to HAMLET, with WiDr cells showing greater resistance, pointing to factors beyond genetic mutations influencing drug responses. A slight synergy between HAMLET and FOLFOX was observed in WiDr cells, independent of the BRAF mutation. The bioenergetic analysis highlighted differences in mitochondrial respiration between HT-29 and WiDr cells, suggesting that bioenergetic profiles could be key in determining cellular responses to HAMLET. *Conclusions:* We highlight the potential of HAMLET and FOLFOX as a combined therapeutic approach in BRAF wild-type CRC, significantly reducing cancer cell viability. The varied responses in CRC cell lines, especially regarding bioenergetic and mitochondrial factors, emphasize the need for a comprehensive approach considering both genetic and metabolic aspects in CRC treatment strategies.

## 1. Introduction

Colorectal cancer (CRC) stands as the third most prevalent neoplasm, with over 1.9 million cases annually, and is the second most lethal, accounting for nearly a million deaths each year [1,2]. Despite advances in diagnostics, screening, lifestyle awareness, and combined therapeutic regimens that have elevated the 5-year overall survival rate to 65% [3], the 5-year survival rate drastically drops to 13% for patients who either present with distant disease at diagnosis or develop it later [4,5].

A deeper understanding of the biology and heterogeneity of CRC is the next challenge for precision medicine. Researchers intend to categorize CRC based on genetic and molecular markers [6].

A notable molecular variation, the BRAF V600E mutation (mt), prevalent in 8–12% of CRC patients, is linked to a poor prognosis and diminished treatment responsiveness. This mutation, often associated with alterations in the MAPK pathway and the tumor environment, frequently coexists with other molecular markers, thus complicating therapeutic approaches [7,8].

Numerous studies consistently suggest that patients whose tumors carry the BRAF V600E mutation show reduced response rates to EGFR inhibitors, regardless of whether these drugs are administered alone or alongside chemotherapy. This diminished efficacy remains evident even in tumors with a RAS wild-type (wt) status, underlining the therapeutic challenges specific to the BRAF V600E mutation. For those with BRAF V600E mutant mCRC, FOLFOX- and bevacizumab-based chemotherapy is still the preferred initial treatment strategy [9,10,11,12].

In the ongoing search for innovative therapeutic agents, particularly for challenging cases of CRC, the HAMLET complex (human alpha-lactalbumin made lethal to tumor cells) has emerged as a potential game-changer. Derived from components of human breast milk, HAMLET has a unique ability to selectively target cancer cells while leaving healthy cells unharmed [13,14]. This is critical in BRAF mutant CRC, which often resists standard therapies [15]. Our previous findings with HAMLET have demonstrated a pronounced different effect on the BRAF mutant cell line than others, revealing its dose-dependent cytotoxic effects on CRC cells, predominantly leading to necrotic death, and altered mitochondrial functions in specific cell lines [16].

A breakthrough approach in CRC research involves ex vivo patient biopsies, where tumor tissues are cultivated outside the body to assess treatment responses. This method offers a window into individual tumor drug sensitivity and emphasizes the heterogeneity intrinsic to CRC tumors [17]. However, maintaining intrinsic tumor characteristics in an ex vivo environment presents significant challenges. With the increasing emphasis on precision medicine, it is imperative to comprehend the advantages and disadvantages of ex vivo patient biopsies in treating CRC [18,19].

In light of our initial observations, we aimed to evaluate the effects of combining HAMLET with the standard chemotherapy FOLFOX on BRAF mutant CRC cell lines and ex vivo explant models to assess whether the response to HAMLET is driven by the BRAF mutation or by mutation-independent mechanisms such as mitochondrial function.

## 2. Materials and Methods

### 2.1. Patient Cohort and Explant Formation of Human Colorectal Cancer Biopsy

We included adult patients diagnosed with colorectal cancer who underwent surgery at the Hospital of the Lithuanian University of Health Sciences Kaunas Clinics between 2021 and 2022. Each patient provided informed consent, and this research received full ethical approval from the Kaunas Regional Biomedical Research Ethics Committee (approval number BE-2-64, dated 1 August 2019). All procedures performed in this study complied with the relevant regulations following the Declaration of Helsinki.

**Chart 1:**
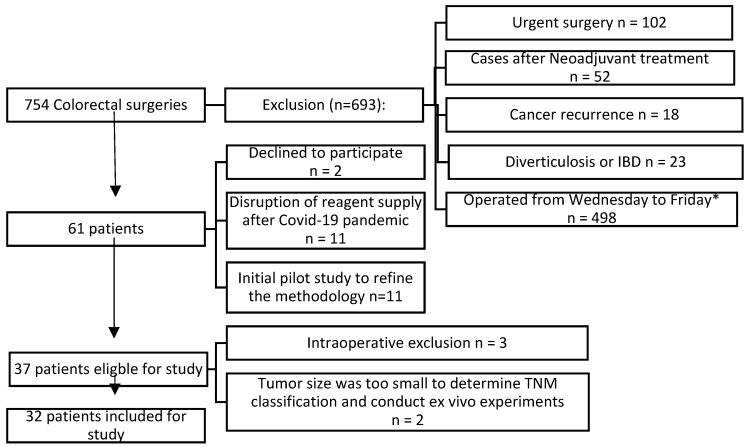
Overall flow chart design of colorectal surgeries in 2021 and 2022 and inclusion for study. IBD—inflammatory bowel disease. * We only included patients operated on Monday or Tuesday due to methodology.

During the specified time period, 754 colorectal surgeries were performed at our institution, of which a selected group of 32 cases met the specific inclusion criteria of this study. The strict parameters of our selection process required us to exclude surgeries performed from Wednesday to Friday to coincide with the timing of ex vivo experimental evaluations, as well as emergency surgeries, cases of recurrent cancer, and cases involving neo-adjuvant therapy, diverticulosis, or inflammatory bowel disease (IBD). Our methodology was further refined by omitting cases from the initial pilot study, accounting for patients who declined to participate, and adjusting for a pause in this study due to the impact of the COVID-19 pandemic on reagent availability. The study design is detailed in Chart 1.

After colorectal cancer resection surgery, pathologists evaluated the excised section of the large bowel. After assessing the tissue and determining the localization of the tumor, a piece of the tumor was cut and placed in a cold cell culture medium (minimal essential medium (MEM)) with 10% fetal bovine serum (FBS) and antibiotics/with a mixture of antimycotics. A piece of tissue was brought to a vertical flow laminar, and, furthermore, all manipulations were performed under sterile conditions with sterile tools and reagents.

Since the large intestine contains various intestinal bacteria and the excised part of the intestine was evaluated under non-sterile conditions, the tissue inevitably became infected with various bacteria and fungi. To stop the growth of bacteria or fungi but not harm the cells, the nutrient medium was supplemented with commonly used and non-toxic doses of antibiotics: penicillin/streptomycin and amphotericin B solution, metronidazole, cefuroxime, and gentamicin, as well as 10% FBS.

**Figure 1:**
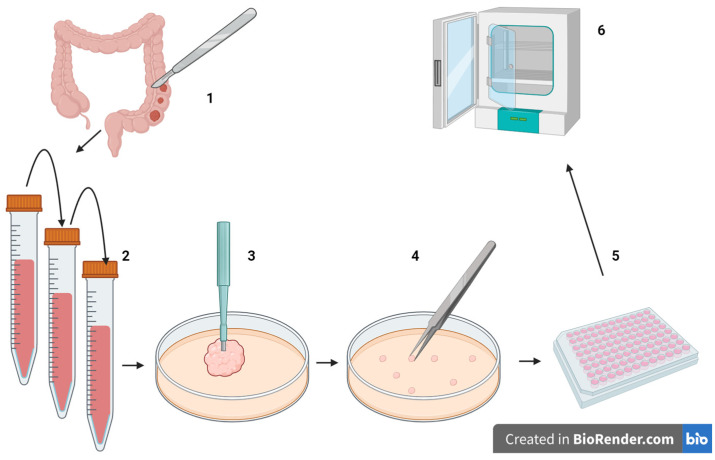
Establishing the explant model: 1—a piece of tumor and healthy tissue is collected after operation and examination; 2—the pieces are washed three times under sterile conditions; 3—the pieces are cut into 2 mm pieces with a biopsy needle, avoiding shredding as much as possible; 4—we are left with 2 mm^3^ explants; 5—every single explant is placed into a well of 96-well plate; and 6—incubation at 37 °C, 95—98% humidity, and 5% CO_2_ atmosphere.

The piece of tissue was washed three times with a nutrient medium to remove as many microorganisms and blood cells as possible. After washing, the tissue was cut with 2 mm diameter biopsy needles, trying to avoid tearing the tissue. Colon cancer pieces of a uniform size of approximately 2 mm^3^ were obtained. Each piece was individually placed in a well of a 96-well plate with a nutrient medium and incubated for 24 h. The pieces were incubated in an incubator that maintained a temperature of 37 °C, 95–98% humidity, and a 5% CO_2_-saturated environment (Figure 1).

### 2.2. Mutation Analysis—RT PCR

Genomic DNA was extracted from CRC tissue using a DNeasy Blood and Tissue kit (Qiagen, Hilden, Germany). Purified DNA was quantified and assessed for purity using UV spectrophotometry. For BRAF V600E mutation detection, we used predesigned TaqMan assays (Life Technologies, Carlsbad, CA, USA) (rs113488022) with a TaqMan Universal PCR Master Mix, No UNG (Life Technologies, Carlsbad, CA, USA) and an approximately 20 ng per well DNA sample. Amplification was performed using the ABI 7500 fast Real-Time PCR system. Genotype assignments were manually confirmed via visual inspection with the ABI 2.3 software compatible with the TaqMan^®^ system. After initial genotyping, 25% of all samples in each group were included in a repetitive analysis, which showed a 100% concordance rate.

### 2.3. Cell Cultures

We purchased the WiDr (colorectal adenocarcinoma) cell line from CLS Cell Lines Service in Germany and the HT-29 (colorectal adenocarcinoma) cell line from the American Type Culture Collection (ATCC) in the United States. The HT-29 cell line was cultured in McCoy’s 5A medium, supplemented with 10% fetal bovine serum and 1% penicillin–streptomycin, all obtained from GIBCO. The WiDr cell line was maintained in a 1:1 mixture of Ham’s F-12K (Kaighn’s) medium and Dulbecco’s Modified Eagle Medium, enriched with 5% fetal bovine serum and 1% penicillin–streptomycin. Both cell lines were incubated at 37 °C in an atmosphere containing 5% CO_2_.

### 2.4. Formation of the HAMLET Complex

We prepared the HAMLET complex by combining human alpha-lactalbumin with oleic acid (obtained from Sigma-Aldrich in Steinheim, Germany), following the heat treatment method outlined in the literature [20]. We first dissolved human alpha-lactalbumin in phosphate-buffered saline and then shook the solution at 50 °C for 15 min. Subsequently, we added oleic acid to the mixture and continued the incubation with shaking for another 10 min at the same temperature. We allowed the solution to cool down to room temperature before centrifuging to remove any excess oleic acid. Finally, we stored the HAMLET complex at −80 °C for future use.

### 2.5. Explant Treatment with HAMLET

After 24 h of incubation, 60 μM of HAMLET was added into the appropriate wells, and the plate was incubated for 24 h. The dose was chosen according to previous experiments with explants, which showed a statistically significant effect only when treated with 60 μM of HAMLET for 24 h. Subsequently, the medium was changed to a medium supplemented with 10% resazurin. The explant metabolizes the purple-colored compound, resazurin, into a pink-colored compound, resorufin, which can be measured with a spectrometer using 570 nm and 620 nm filters. Since resazurin is a non-toxic compound, the measurements were made 24 and 48 h after HAMLET treatment.

Alternatively, after a 24 h treatment with HAMLET, the explant samples were collected, and the oxygen consumption and extracellular acidification rates were measured.

### 2.6. Mitochondrial Respiration

We studied the mitochondrial respiration, specifically, oxygen consumption, of ex vivo tissue samples and two colon cancer cell lines harboring BRAF mutations (HT-29 and WiDr) using the high-resolution respirometry system Oxygraph-2k (OROBOROS Instruments, Innsbruck, Austria) at 37 °C. The medium for the measurements contained 0.5 mM of EGTA, 3 mM of MgCl_2_, 60 mM of K-lactobionate, 20 mM of taurine, 10 mM of KH_2_PO_4_, 20 mM of HEPES, and 110 mM of sucrose, adjusted to pH 7.1 at 37 °C. To permeabilize the cell membranes, 16 μg/mL of digitonin was added. We documented the non-phosphorylating state 2 (V0) respiration rate in the medium containing the tissue samples, mitochondrial complex I substrates (5 mM of glutamate + 2 mM of malate), and the complex II substrate succinate (12 mM). After adding 1 mM of ADP, we assessed the state 3 respiration rate (VADP). All respiration rates were normalized to the dry weight of the tissue in milligrams.

### 2.7. Cell Viability Flow Cytometric Analysis

We used the MTT colorimetric assay (obtained from Thermo Fisher Scientific and Carl Roth in Steinheim, Germany and Karlsruhe, Germany) to assess cell viability and the cytotoxic effects of HAMLET over 48 h. We initially seeded cells into 96-well plates at densities appropriate for each cell line, ranging from 8000 to 20,000 cells per well. After 24 h, we treated the cultures with the HAMLET complex and incubated them for another 6 h. We then replaced the growth medium and allowed the cells to incubate for another 18 h before adding the MTT reagent. After incubation for 3–4 h at 37 °C, we dissolved the formazan crystals in dimethyl sulfoxide, measured the absorbance at 570/620 nm, and compared it with a control group.

### 2.8. Clonogenic Assay

For the clonogenic assay, we plated cells at a density of 100 to 200 cells per well in 24-well plates. After allowing 24 h for attachment, we administered various concentrations of the HAMLET complex for 6 h. Then, we changed the medium and incubated the cells for 8 days. After incubation, we fixed and stained the colonies with crystal violet and counted those with more than 50 cells using an inverted microscope and compared the results with controls.

### 2.9. Flow Cytometric Analysis

We used the Flow Cellect Mito Damage Kit and Annexin V-PE Apoptosis Detection Kit obtained from EMD Millipore in the United States. To perform the flow cytometric analysis and understand how cells responded to HAMLET, we seeded 100,000 to 130,000 cells per well. After one day, we treated these cells with different concentrations of HAMLET for 6 h. We then detached the cells, stained them with Annexin V-PE and 7-AAD, and analyzed them using the Guava Personal Cell Analysis Flow Cytometer and CytoSoft software (version 2.1.4; Guava; EMD Millipore, Burlington, MA, USA).

### 2.10. Drug Combination Effect Calculation

The combination effect of the HAMLET complex and FOLFOX (5-fluorouracil (5-FU) + oxaliplatin) was measured via MTT assay and calculated using the Combenefit (v2.021) software [21]. Bliss theory was chosen for the calculation theory based on the assumption that both drugs work independently but can increase each other’s cytotoxic effects [22]. Varying doses of HAMLET (1, 3, and 5 μM) and FOLFOX (5-FU (μM) + oxaliplatin (μM): 3.125 + 0.078; 6.25 + 0.156; 12.5 + 0.3125; 25 + 0.625; 50 + 1.25; 100 + 2.5; 200 + 5; 400 + 10; and 800 + 20) were used.

### 2.11. Glycolytic and Mitochondrial Activity Determination

The mitochondrial and glycolytic activity of WiDr and HT-29 cells was measured with a Seahorse XFp Analyzer (Agilent Technologies, Santa Clara, CA, USA) using the Seahorse XFp Cell Mito Stress Test Kit (Agilent Technologies) according to the manufacturer’s instructions. All the assay and data interpretation details are available in the User Guide [23]. Briefly, the cells were seeded into Agilent Seahorse XFp miniplates at a density of 1.5 × 10^3^ to 3 × 10^3^ cells/well and kept in the cell culture medium indicated above. The cells were incubated for 4 d until 50–80% confluency was reached. One hour before the measurement, the medium was replaced with Seahorse XF Assay Medium supplemented with 2 mmol/L of L-glutamine, 1 mmol/L of sodium pyruvate, and 10 mmol/L of glucose, and the cells were placed in a non-CO_2_ incubator. Just before the measurement, the medium was changed again to fresh Assay Medium with the same supplements. The final inhibitor concentrations in the wells were 1.5 μM oligomycin, 1 μM carbonyl cyanide-4-phenylhydrazone, 0.5 μM antimycin A, and 0.5 μM rotenone. The oxygen consumption rate (OCR) and extracellular acidification rate were normalized to the total cellular protein content determined directly in the plate using the Bradford assay. The data were analyzed using Wave software 2.6.1 (Agilent Technologies), and graphical images were created employing SigmaPlot vs. 13 (Systat Software, Slough, UK).

### 2.12. Statistical Analysis

We carried out our statistical analyses using GraphPad Prism 6 with SigmaPlot. To analyze non-parametric datasets, we applied the Mann–Whitney *U* test. We examined the correlations between qualitative measures in comparative cohorts using the chi-square (χ^2^) test, while Student’s *t*-test was employed for interval and categorical data assessments. The significance level was set at *p* < 0.05.

## 3. Results

### 3.1. Colorectal Cancer Patients’ Explant Characteristics

We analyzed 32 patients with colorectal carcinoma (mostly moderately differentiated, G2 (84.4%)). The mean age of CRC patients was 68.06 ± 11.95, with a gender distribution of 21 females and 11 males. BRAF mutations were identified in four patients, accounting for 12.5% of the cohort. The serum markers CEA and CA 19-9 were predominantly below normal limits in 65.6% and 87.5% of patients, respectively. The primary site of cancer was predominantly the rectum, with six patients undergoing neoadjuvant treatment. A detailed analysis of the TNM staging, postoperative Clavien–Dindo complications, and patient follow-up data are outlined in Table 1.

**Figure 2:**
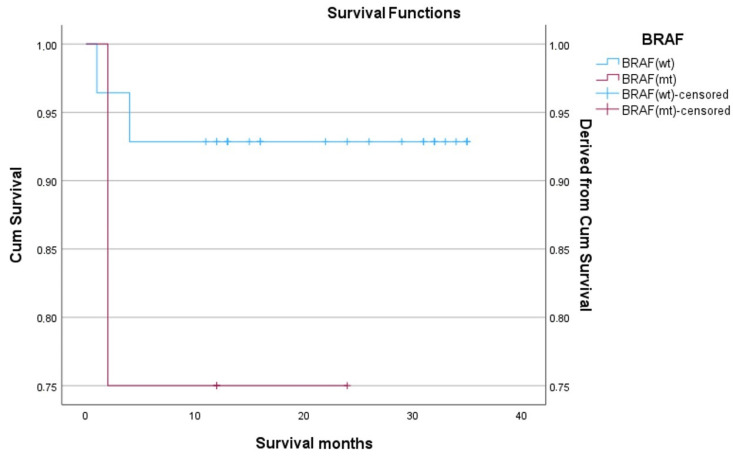
Kaplan–Meier analysis by month, stratified by mutation status.

In our Kaplan–Meier survival analysis (Figure 2), we observed that patients with BRAF wt mutations had a longer median survival of 32.68 ± 1.58 months (95% CI: 29.58–35.78) compared with 18.5 ± 4.76 months (95% CI: 9.16–27.84) in patients with BRAF mutant types. However, the difference in the survival curves was not statistically significant (*p* = 0.247). Notably, follow-up was longer in the BRAF wt group (22.29 ± 10.53 months; range: 1–35) than in the BRAF mt group (14.42 ± 7 months; range: 2–24).

### 3.2. Colorectal Cancer Explant Viability

In our study, we observed that both HAMLET and FOLFOX, when used alone, exerted similar cytotoxic effects on CRC explants, regardless of the BRAF mutation status. Specifically, HAMLET reduced the viability of both wild-type and mutant BRAF explants to approximately 87% at 24 h and 86% wt and 84% mt at 48 h. Similarly, FOLFOX treatment reduced the viability of the BRAF wt explants to 85% at 24 h and 79% at 48 h, while the mt explants showed a comparable reduction to 78% at 48 h.

**Figure 3:**
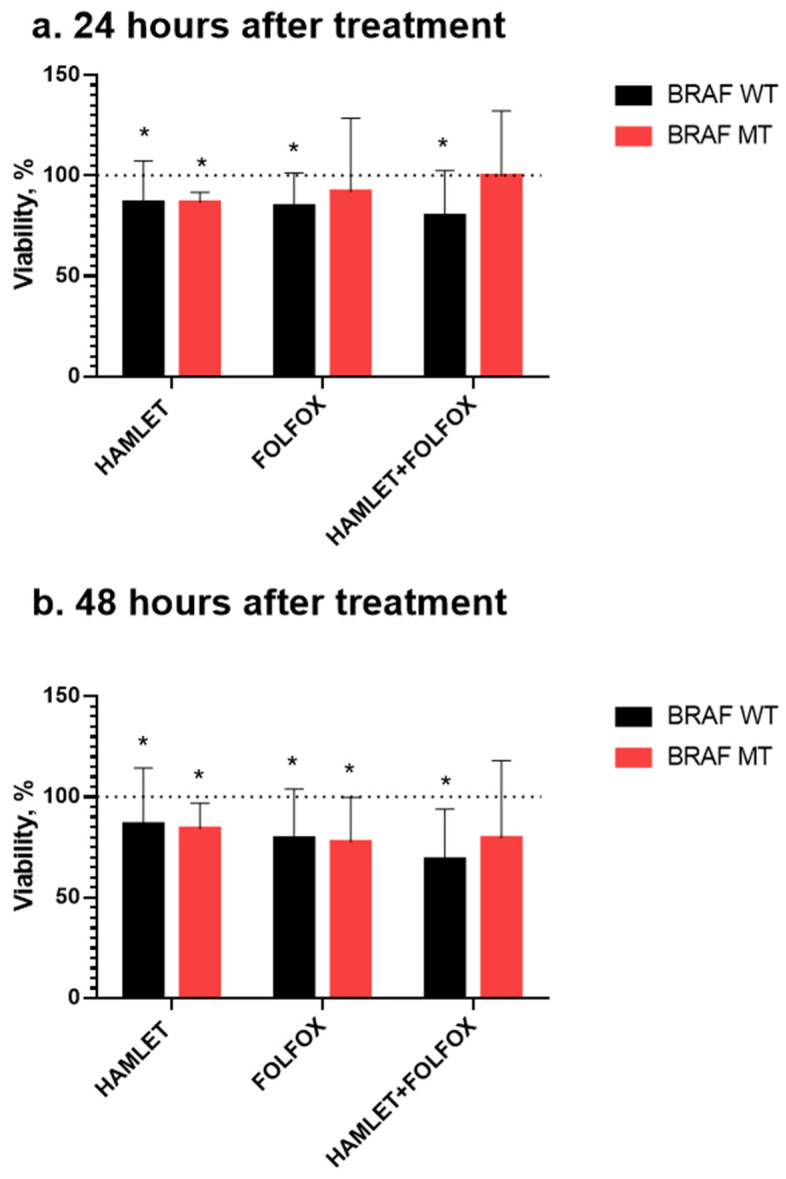
The effect of 60 μM of HAMLET and/or FOLFOX (3 mM of 5-FU + 75 μM of oxaliplatin) on CRC explant viability. (**a**) Explant viability 24 h after treatment; (**b**) explant viability 48 h after treatment. Comparing BRAF wild-type and mutant explants. Means ± SD. N ≥ 3. Dotted line—control group data (100%). *—*p* < 0.05 comparing with control group data.

However, the combination of HAMLET and FOLFOX showed a more pronounced effect on the BRAF wt group. This combined treatment reduced the viability of BRAF wt explants to 80% at 24 h and to 69% at 48 h, indicating a synergistic effect. In contrast, the same combination did not significantly alter the viability of BRAF mt explants (Figure 3).

### 3.3. Explant Mitochondrial Respiration

In our investigation of mitochondrial respiration in human CRC tissue explants, we evaluated the impact of HAMLET (60 μM) on mitochondrial function. Our assays focused on measuring the mitochondrial respiration rate at 37 °C, utilizing glutamate/malate (Complex I) and succinate (Complex II) as substrates. We observed that HAMLET tended to inhibit mitochondrial respiration in both BRAF mutant and wild-type CRC tissue samples, though this inhibition was not statistically significant (*p* > 0.05).

**Figure 4:**
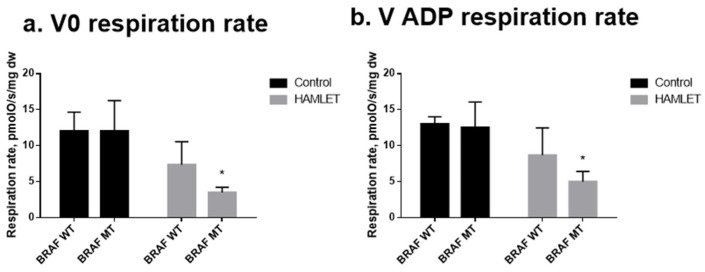
Comparison of the impacts of HAMLET and control on mitochondrial functions between BRAF wild type and BRAF mutant: (**a**) mitochondrial non-phosphorylating (V0) respiration rate and (**b**) mitochondrial state 3 (VADP) respiration rate are means ± SD. *—*p* < 0.05 comparing non-treated control and HAMLET-treated samples.

Specifically, in CRC samples without the BRAF mutation, HAMLET treatment led to a 39% reduction in the non-phosphorylating (V0) respiration rate (from 12 ± 2.646 to 7.3 ± 3.215 pmolO/s/mg dry weight) and a 33.3% decrease in the state 3 (VADP) respiration rate (from 13 ± 1 to 8.67 ± 3.786 pmolO/s/mg dry weight). In contrast, tissue samples with the BRAF mutation exhibited a more substantial impact, with a 71% reduction in the V0 respiration rate (from 12 ± 4.243 to 3.5 ± 0.7 pmolO/s/mg dry weight) and a 60% decrease in the VADP respiration rate (from 12.5 ± 3.54 to 5 ± 1.41 pmolO/s/mg dry weight). These findings suggest a differential response in mitochondrial respiration inhibition by HAMLET between BRAF mt and wt CRC samples, with a greater extent of inhibition observed in the BRAF mt samples (Figure 4b).

### 3.4. Comprehensive Analysis of HAMLET Complex Effects on CRC BRAF Mutant Cells: Viability, Clonogenic Survival, and Induced Apoptosis/Necrosis

We then investigated why the BRAF mutant has distinctive features in viability and respiration studies. Therefore, we chose two genetically identical colon cancer cell lines with the BRAF mutation.

**Figure 5:**
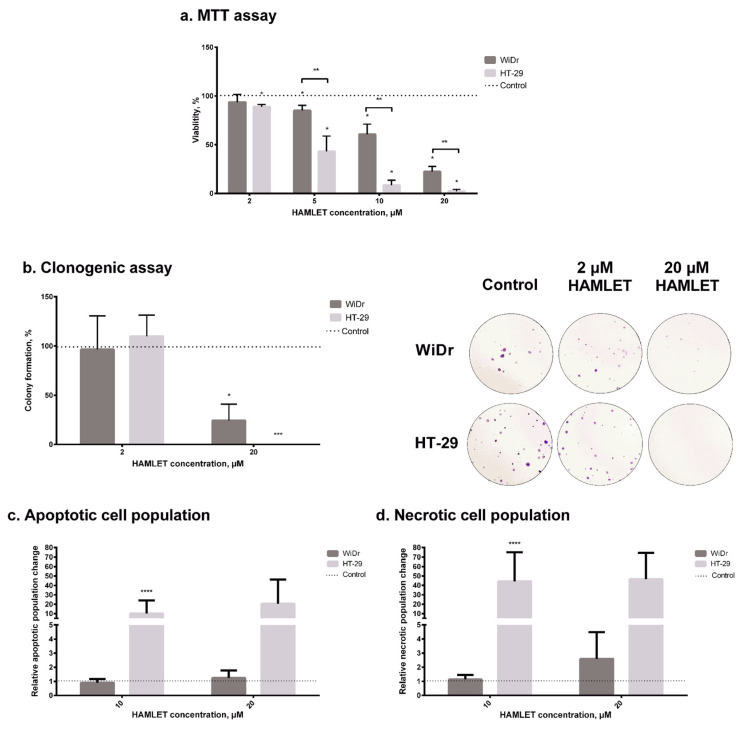
Dose-dependent responses of CRC cell lines to HAMLET complex. (**a**) The MTT assay performed 24 h after a 6 h incubation revealed a dose-dependent response and higher WiDr cell line resistance to HAMLET than HT-29. (**b**) The effect of HAMLET on colony formation in different BRAF mutant CRC cell lines. Graphic representation and representative photos. (**c**) Apoptotic cell line population after the effect of HAMLET. (**d**) Necrotic cell line population after the effect of HAMLET. Control group data—dotted line. * *p*  <  0.05—compared with control group data. ** *p*  <  0.05—comparing HAMLET’s effects on WiDr and HT-29. ***—no colony formation. **** *p*  ≤  0.05 when comparing apoptosis and necrosis of the same sample. Means ± SD. N ≥ 3.

In our evaluation of the effects of the HAMLET complex on different CRC cell lines (HT-29 and WiDr (a derivative of HT-29)) with V600E BRAF mutation status, we found consistent dose-dependent effects on viability at 2 μM, 5 μM, 10 μM, and 20 μM concentrations. At 2 μM, the viability of the WiDr cell line remained largely unaffected, while the HT-29 line experienced an 11% reduction in viability to 89%. As the concentration increased, the differences in resistance between the two lines became more pronounced: At 5 μM, WiDr retained 85% viability compared with 43% for HT-29. At higher concentrations of 10 and 20 μM, WiDr’s viability dropped to 61% and 22%, respectively, while HT-29’s viability dropped to 8% and 2% (Figure 5a).

Further analysis, focusing on clonogenic potential, showed that while 2 μM of the HAMLET complex showed no discernible reduction in colony formation for either cell line, a drastic effect was observed at 20 μM. Herein, the colony formation of the WiDr line dropped to 24%, while the HT-29 line showed a complete loss of its ability to form colonies (Figure 5b).

Flow cytometry confirmed these findings (Figure 5c,d), with a marked shift toward apoptosis and necrosis after HAMLET treatments. The apoptotic cell population of WiDr decreased by 10% at 10 μM and increased 1.25-fold at 20 μM, whereas HT-29 showed a dramatic increase: 10-fold and 20-fold at 10 μM and 20 μM, respectively. The shift toward necrosis was even more pronounced, especially for HT-29, which showed staggering 44-fold and 47-fold increases at these concentrations.

However, in all these assays, WiDr consistently showed greater resistance. Taking these observations together (Figure 5a–d), it is clear that WiDr’s resistance to HAMLET far exceeds that of the HT-29 line despite having identical mutations. Such results indicate that the response to the HAMLET complex may be controlled by factors beyond mere genomic or mutational characteristics, prompting us to investigate the different energetics of these cell lines.

### 3.5. Bliss Synergy Model Calculation

**Figure 6:**
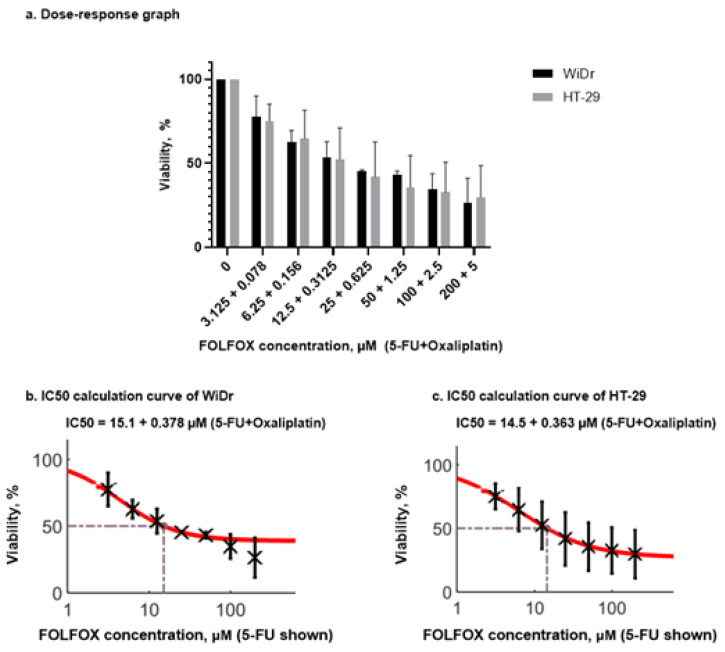
Cell responses to FOLFOX. (**a**) Dose–response graph of WiDr’s and HT-29′s responses to different doses of FOLFOX. (**b**) FOLFOX IC50 dose calculation for WiDr cell line. (**c**) FOLFOX IC50 dose calculation for HT-29 cell line. N = 3; means ± SD. Purple dot marks 50% viability (IC50).

Opposite to their responses to HAMLET, both cell lines showed similar tendencies when treated with FOLFOX. They responded to FOLFOX in a dose-dependent manner (Figure 6a). When calculating the IC50 doses of both cell lines, there was a minimal difference between them, with WiDr’s IC50 dose being 15.1 + 0.378 μM and HT-29’s being 14.5 + 0.363 μM (5-FU + oxaliplatin) (Figure 6a,b).

**Figure 7:**
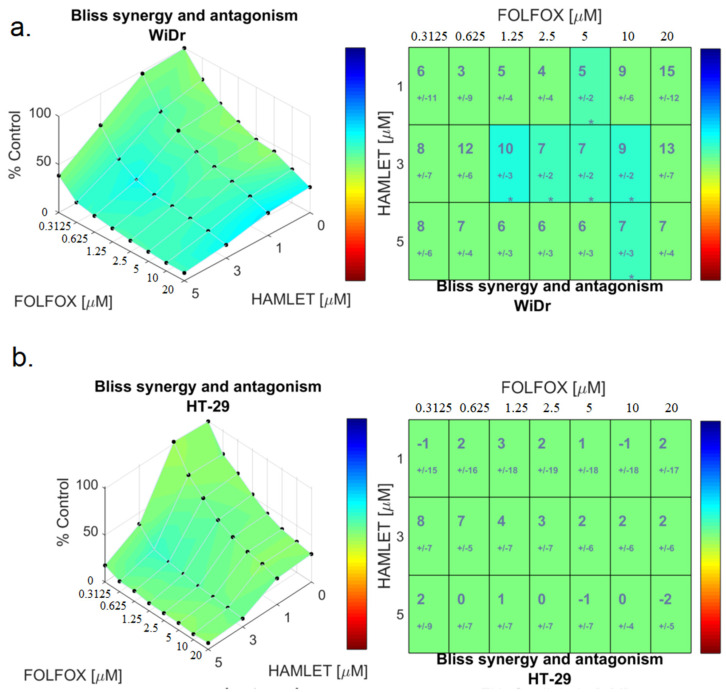
Heatmaps and tables of Bliss synergy and antagonism calculation. (**a**) WiDr cell line; (**b**) HT-29 cell line. Heatmaps show decrease in viability when treating with varying doses of FOLFOX and/or HAMLET compared with control (100% viability). Tables show synergy or antagonism index. Higher positive number with blue color shows significant synergy, and negative number with red color shows significant antagonism (not present). *—*p* < 0.05.

The results of the commonly used chemotherapy drug combination of FOLFOX and the HAMLET complex showed a slight synergy between FOLFOX and HAMLET but only in the WiDr cell line. The highest significant synergy score obtained was when treating cells with 3 μM of HAMLET + 1.25 of FOLFOX (50 μM of 5-FU + 1.25 μM of oxaliplatin). However, most of the combinations of 3 μM of HAMLET and FOLFOX showed a synergistic tendency, while most of the combinations with 1 or 5 μM of HAMLET + FOLFOX showed an additive tendency (Figure 7). The results suggest that these two drugs do not interfere with each other and, in some cases, could even exert a synergistic cytotoxic effect on the cells, which does not depend on the BRAF mutation.

### 3.6. Glycolytic and Mitochondrial Activity Determination

**Figure 8:**
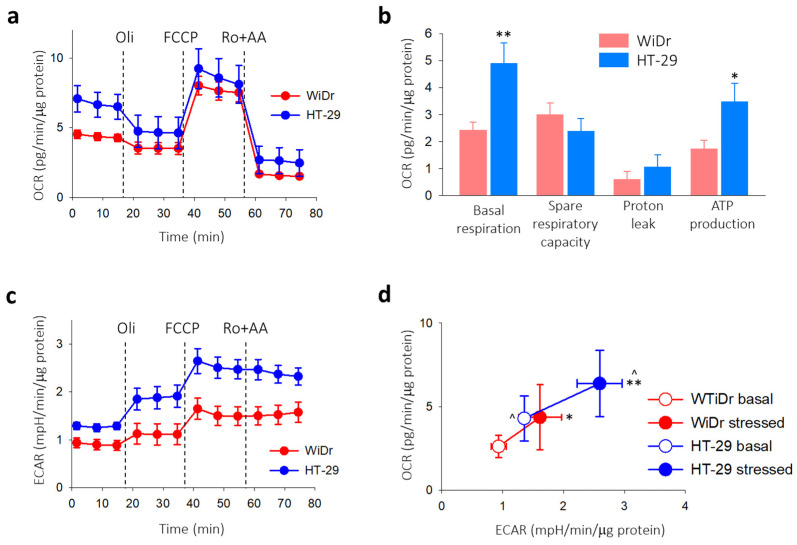
Comparison of the mitochondrial and glycolytic activity of WiDr and HT-29 cells. In (**a**), mitochondrial oxygen consumption curves are presented as averages ± standard deviations of each measurement time point (*n* = 3 of 3 technical replicates). In (**b**), summarized mitochondrial capacity data are calculated from the curves in (**a**). * and **—statistically significant differences compared with WiDr; *p* < 0.05 and *p* < 0.01, respectively. In (**c**), glycolytic activity is monitored as pH changes simultaneously with the oxygen consumption rate. In (**d**), energy phenotype plots represent mitochondrial and glycolytic energy capacity distributions under normal (basal) and stressed conditions. * and **—statistically significant differences compared with basal conditions for the same cell type; *p* < 0.05 and *p* < 0.01, respectively. ^—statistically significant difference compared with the same parameter of WiDr; *p* < 0.05. OCR—oxygen consumption rate, ECAR—extracellular acidification rate, Oli—oligomycin, FCCP—carbonyl cyanide 4-(trifluoromethoxy) phenylhydrazone, Ro—rotenone, and AA—antimycin A.

The observations in Figure 5 show a remarkable divergence in cell lines despite sharing a similar BRAF mutation profile. This prompted an investigation into whether variations in energetic metabolism could account for these differences. Therefore, we compared the cell lines with BRAF mutations for their mitochondrial and glycolytic activity to determine if this was the case. To test the hypothesis, we selected the WiDr and HT-29 cell lines with the same mutational status but different sensitivity to the HAMLET complex and compared their mitochondrial and glycolytic activity. The data of the bioenergetic analysis are presented in Figure 8. The basal mitochondrial respiration of HT-29 cells was significantly higher than that of WiDr cells (the first three points in the oxygen consumption curves in Figure 8a). However, after the addition of the ATP synthase inhibitor oligomycin (to reveal the proton-leak-stimulated OCR), carbonyl cyanide-4-phenylhydrazone (to discover the maximal respiratory capacity), and the respiratory chain complexes I and III inhibitors rotenone and antimycin A (to assess the non-mitochondrial oxygen consumption), there were no significant differences between the respiration rates of WiDr and HT-29 cells. To summarize the results, the WiDr cell line showed a higher resistance to HAMLET than the HT-29 cell line. Moreover, the cellular response to HAMLET was influenced by factors independent of the genomic characteristics of the cells or their mutational status, leading us to characterize the two cell lines by their bioenergetic properties for further insights.

Further analysis of mitochondrial function revealed a significant increase in the basal mitochondrial OCR of HT-29 cells compared with the WiDr line (Figure 8b). The average OCR in HT-29 was about two-fold higher than in WiDr. Similarly, the oligomycin-sensitive or mitochondrial-ATP-production-coupled OCR was two-fold lower in WiDr cells. However, the spare respiratory capacity detected after permeabilizing the inner membrane for H^+^ with carbonyl cyanide-4-phenylhydrazone and proton-leak-driven respiration was not significantly different in WiDr and HT-29 cells.

The glycolysis activity of the cells was assessed as the extracellular acidification rate simultaneously with the OCRs in the identical probes. The basal glycolytic activity in HT-29 cells was significantly higher than that in WiDr, and the difference became even more prominent after each addition of mitochondrial inhibitors (Figure 8c). In addition, cell energy phenotype analysis indicated that unstressed HT-29 cells had the same glycolytic activity as WiDr under maximal mitochondrial stress conditions (Figure 8d). Thus, the more intensive glycolytic response to mitochondrial failure indicated that HT-29 cells had a higher capacity to maintain energetic balance. On the other hand, the sensitive regulation of bioenergetic metabolism might point to the elevated energetic demands of the HT-29 cells, possibly explaining their higher sensitivity to HAMLET treatment.

### 3.7. Mitochondrial Respiration

**Figure 9:**
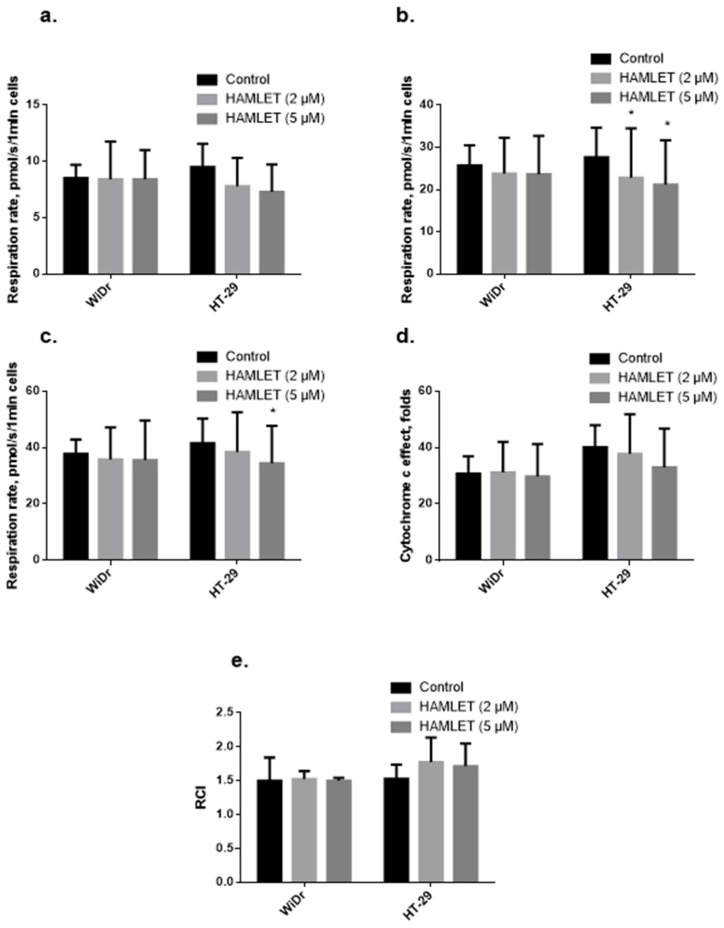
Effect of HAMLET on mitochondrial respiration and respiratory control index (RCI). Mitochondrial respiration rate was measured as described in “Methods”. (**a**) Mitochondrial non-phosphorylating (V_0_) respiration rate in the presence of 1 mln/mL of cells and glutamate (5 mM) plus malate (2 mM); (**b**) state 3 respiration rate in the presence of ADP (1 mM; VADP); (**c**) mitochondrial maximal respiration rate in the presence of succinate (12 mM; Vsucc); (**d**) mitochondrial respiration rate in the presence of cytochrome c (32 μM; Vcyt c); and (**e**) mitochondrial respiratory control index (RCI) (VADP/V0)). * *p*  <  0.05 compared with the control group.

We conducted experiments to assess HAMLET’s impact on mitochondrial respiration in HT-29 and WiDr cancer cells, displaying similar BRAF mutant genetic characteristics. Using glutamate/malate for complex I and succinate for complex II as substrates, we measured the mitochondrial respiration rates at 37 °C. We found that HAMLET, at doses of 2 and 5 μM, did not modify the non-phosphorylating (V0) respiration rate in either cell type, as indicated in Figure 9a. However, HAMLET significantly diminished the state 3 (VADP) respiration rate in HT-29 cells by 17% at 2 μM and by 23% at 5 μM, while it had no such effect on WiDr cells, as depicted in Figure 9b when compared with control cells (*p* < 0.05). In HT-29 cells, pretreatment with HAMLET at 5 μM led to a 17% reduction in the maximal mitochondrial respiration (Vmax) with complex I and II substrates, whereas 2 μM of HAMLET had no significant effect, as shown in Figure 9c against untreated cells (*p* < 0.05). Neither 2 μM nor 5 μM of HAMLET influenced the maximum mitochondrial respiration rate in WiDr cells. Adding cytochrome c to the mitochondrial mix, as seen in Figure 9d, confirmed that HAMLET pretreatment at both concentrations did not impact the mitochondrial respiration rate (Vcyt. c) in either cell line, suggesting no change in mitochondrial outer membrane permeability. Lastly, the respiratory control index (RCI), observed in Figure 9e, remained stable in cells pre-treated with HAMLET at both concentrations compared with the control cells in both the HT-29 and WiDr cell lines.

## 4. Discussion

In a breakthrough approach, researchers at Lund University have pioneered the use of HAMLET as a novel molecular agent for the treatment and prevention of CRC. HAMLET’s ability to selectively accumulate in tumor tissue and its demonstrated efficacy in reducing tumor burden and mortality in Apc(Min)(/+) mice heralds a new era in targeted therapies for CRC [13,24].

In our previous study, we found resistance of BRAF mutant cancer cells to HAMLET [16]. One of the objectives of the current research was to determine whether this mutation is related to the effects of this complex. According to the data from this study, in cell lines with an identical BRAF mutation, the resistance of WiDr to HAMLET is significantly higher than that of the HT-29 line. To date, there has been limited investigation of the interaction between HAMLET and CRC. This gap is further bridged only by the parallel investigation of BAMLET, a similar compound derived from bovine alpha-lactalbumin. Behrouj and colleagues have been investigating the effect of BAMLET on cell survival mechanisms in RAS-mutant HCT116 cells. BAMLET reduces CK1α expression, interferes with key signaling pathways, and inhibits cellular recycling processes, leading to increased cell death, particularly when combined with specific kinase inhibitors [25]. Compared with the BAMLET study cell line, intriguingly, unpublished observations from our laboratory suggest that HCT116 cells, despite having a different mutational profile than HT-29 cells, respond positively to HAMLET, mirroring the response in HT-29. Such findings hint that HAMLET may be broadly applicable across different CRC mutations. However, factors other than genomic or mutational specificities may control the response to the HAMLET complex.

One of the potential factors are mitochondrial functions and bioenergetic analysis. We found that HAMLET’s inhibitory effect on mitochondrial respiration is cell-type-specific within the context of BRAF mutant colorectal cancer cells. The different sensitivities to HAMLET in BRAF mutant cells might be due to differences in their energy metabolism. Specifically, HT-29 cells exhibit significantly higher basal mitochondrial respiration and glycolytic activity compared with WiDr cells. Despite their mutation status, HT-29 cells are more sensitive to HAMLET, which could be linked to their higher energetic demands, as indicated by their more intensive glycolytic response to mitochondrial stress. This suggests that the ability of HT-29 cells to maintain energy balance is greater than that of WiDr cells, which might contribute to their differing responses to HAMLET treatment. The results imply that factors beyond genomic characteristics, such as cellular energy metabolism, play a role in the efficacy of HAMLET against colorectal cancer cells. Only one study has investigated the interaction between HAMLET and mitochondrial respiration. The authors posited that HAMLET induces cell death, highlighting its potential to target and kill tumor cells via a direct effect on their mitochondrial function [26].

Our study confirms the critical role of metabolic profiles in the response of cancer cells to treatments, as previously noted by Lin et al. [27]. Specifically, we found that HT-29 cells, which have higher glycolytic rates than WiDr cells, are more susceptible to HAMLET, a therapeutic protein complex. This susceptibility is based not only on genetic markers but also on distinct metabolic behaviors, a notion supported by Rebane-Klemm et al., who found metabolic phenotype variations in colorectal tumors with KRAS and BRAF mutations [28]. We found that HAMLET selectively impairs mitochondrial respiration in HT-29 cells, even though both HT-29 and WiDr cells share a BRAF mutation, suggesting that the metabolic phenotype dictates treatment sensitivity. This is consistent with the findings of Cha et al. that genetic alterations such as APC loss can drive metabolic changes in cancer cells [29]. Our results differ from the effects of vitamin C on KRAS-mutant colon cancer reported by Cenigaonandia-Campillo et al., suggesting that different treatments may exploit unique metabolic vulnerabilities [30]. In addition, the studies by Spier et al. and Monterisi et al. highlight the role of mitochondrial function in cell fate and survival, emphasizing the interplay between metabolic and genetic factors in cancer therapeutics [31,32]. Kealey et al. also highlight the complexity of cancer cell bioenergetics, as TP53 deficiency and KRAS signaling can alter cellular responses to various substrates, indicating that metabolic responses are multifaceted and context-dependent [33]. Collectively, our findings highlight the importance of an integrative approach that considers both genetic mutations and metabolic features in the development of targeted cancer therapies.

**Figure 10:**
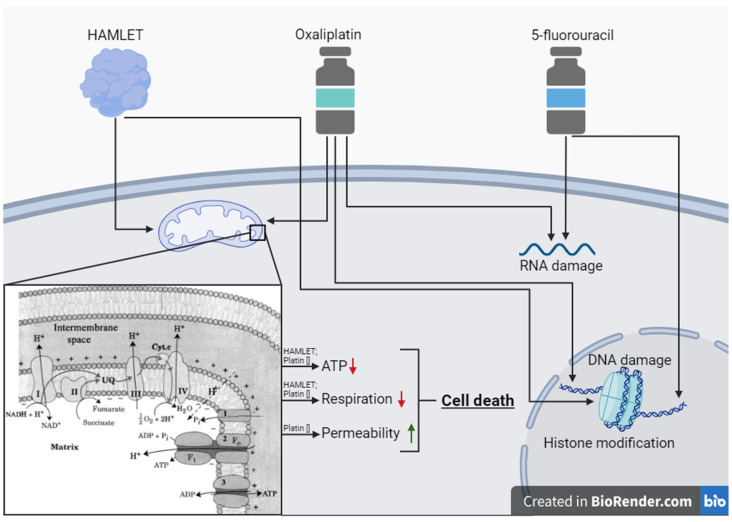
Schematic representation of the possible combined effect of HAMLET and FOLFOX components (oxaliplatin and 5-fluorouracil). The combined effect of FOLFOX component oxaliplatin and HAMLET is most likely exerted via an effect on mitochondria. HAMLET decreases cell ATP production and cell respiration, and our previous studies show that platin-based drugs reduce ATP production, respiration, and mitochondria membrane permeability (Caco-2, AGS, and T3M4 with cisplatin). The combined effect of HAMLET and platin-based drugs on mitochondria can, in some cases, have synergistic effects, leading to increased cell death via mitochondrial damage. ↑ increaced ↓ decreased.

We included a scheme that illustrates the mechanism (Figure 10) where a combination of HAMLET and the components of FOLFOX (oxaliplatin and 5-fluorouracil) induces cancer cell death. The diagram highlights the effect of these agents on mitochondrial function, a critical aspect of this study’s findings. It shows that while HAMLET alone reduces ATP production and cell respiration, its combination with oxaliplatin further disrupts these processes, as demonstrated by our institution’s previous studies with platinum-based drugs in various cancer cell lines or Wei Sun et al.’s study [34,35]. This combined effect impairs mitochondrial function and increases mitochondrial membrane permeability, leading to a synergistic escalation of cell death. The following visual representation clarifies the underlying biochemical interactions and supports this study’s hypothesis of a combined therapeutic effect against CRC cells.

The results of our study on the efficacy of HAMLET in combination with FOLFOX in CRC are consistent with the work of James M.I. et al., who reported that curcumin safely enhances the effects of FOLFOX in a clinical setting [36]. We extend this by showing that the HAMLET–FOLFOX combination selectively reduces viability in BRAF wild-type explants, highlighting the potential for tailored therapies based on genetic profiles and laying the foundation for future clinical applications. For example, a similar bioactive agent to HAMLET is resveratrol in combination with 5-fluorouracil [37], which could be used as an adjunct to conventional chemotherapy.

Our goal is to translate our promising in vitro and ex vivo findings into clinical trials to evaluate the efficacy and safety of this combination therapy in CRC patients. This approach has the potential to improve outcomes, particularly in cases resistant to conventional treatments.

In addition, the diverse responses of CRC cell lines to treatment, which are influenced by mitochondrial dynamics, underscore the need for personalized medicine in CRC therapies [18,19]. Our research suggests that incorporating genetic and metabolic profiling into treatment design could lead to more targeted and effective strategies, with the ultimate goal of improving patient prognoses and treatment responses.

The drawbacks of our research include a limited sample size, which may not reflect the diversity of the broader CRC population, affecting the generalizability of our findings. In addition, we focused on the BRAF mutation and may have overlooked the impact of other mutations on the efficacy of HAMLET [38]. Moreover, keeping the biopsy tissue alive and maintaining its native characteristics outside the body is challenging. Finally, while informative, our in vitro and ex vivo models cannot fully replicate the complex biology of human CRC, including the tumor microenvironment and metabolic effects, which are key to clinical translation. For instance, the examination of intra-tumoral heterogeneity addresses the challenge of differential responses within different cancer cell subpopulations within a single tumor [39]. Further investigations with more comprehensive models are required to validate our findings.

To summarize, our research supports the concept that personalized medicine, tailored to individual genetic and metabolic profiles, may improve outcomes in CRC. Despite this study’s limitations, HAMLET, a combination therapy component for CRC, paves the way for future research that will hopefully translate these findings into clinical practice, offering new hope for targeted, effective treatments for patients with this challenging disease.

## 5. Conclusions

Our study demonstrates that HAMLET and FOLFOX together significantly lower the viability of BRAF wild-type CRC explants via a synergistic effect. HAMLET also moderately inhibits mitochondrial respiration in these cancer tissues. We observed varied responses to HAMLET in CRC BRAF mt cell lines, with WiDr cells being more resistant than HT-29 cells, highlighting the influence of bioenergetic and mitochondrial factors on drug responses. The synergy between HAMLET and FOLFOX in WiDr cells underscores the potential of combined therapies, emphasizing the need to consider both genetic and metabolic aspects in CRC treatment.

## Figures and Tables

**Table 1:** Colorectal cancer patient clinicopathological characteristics. CEA—carcinoembryonic antigen; CA19-9—carbohydrate antigen 19-9, and APR—abdominoperineal resection.

Patient Demographics	N (%)
Gender	Female, 21 (65.6%) Male, 11 (34.4%)
Age at diagnosis	<60 years, 5 (15.6%) ≥60 years, 27 (84.4%)
BRAF mutation status	Wild type, 28 (87.5%) BRAF mutant, 4 (12.5%)
CEA	Normal <5.8, 21 (65.6%) Elevated ≥5.8, 11 (34.4%)
Ca 19-9	Normal <37, 28 (87.5%) Elevated ≥37, 4 (12.5%)
Localization	Right colon, 8 (25.0%) Left colon, 8 (25.0%) Rectum, 16 (50.0%)
Surgery type	Right hemicolectomy, 9 (28.1%) Left hemicolectomy, 2 (6.2%) Sigmoid resection, 6 (18.8%) Rectal resection, 11 (34.4%) APR, 4 (12.5%)
Tumor differentiation	Well-differentiated G1, 3 (9.4%) Mod. differentiated G2, 27 (84.4%) Poorly differentiated G3, 2 (6.2%)
TNM stage	Stage I, 7 (21.9%) Stage II, 15 (46.9%) Stage III, 7 (21.9%) Stage IV, 3 (9.4%)
pT stage	pT1, 1 (3.1%) pT2, 9 (28.2%) pT3, 21 (65.6%) pT4, 1 (3.1%)
pN Stage	pN0, 20 (62.5%) pN1, 10 (31.3%) pN2, 2 (6.2%)
V—vascular invasion	(−) 24 (75.0%) (+) 8 (25.0%)
L—invasion into lymphatic vessels	(−) 25 (78.1%) (+) 7 (21.9%)
Clavien–Dindo postoperative complications at 30-day follow-up	Grade 0 (no complications), 23 (71.9%) Grade I, 1 (3.1%) Grade II, 3 (9.4%) Grade IIIA, none Grade IIIB, 4 (12.5%) Grade IV, none Grade V, 1 (3.1%)
Median postoperative hospital stay	7 days (range: 3–27 days)
Follow-up months	21.06 mo ± 10.73 (min. 1–max. 35)

## Data Availability

The data presented in this study are available on request from the corresponding author.
